# Non-diabetic glycosuria as a diagnostic clue for acute tubulointerstitial nephritis in patients with azotemia

**DOI:** 10.1080/0886022X.2020.1824923

**Published:** 2020-09-27

**Authors:** Taeyeon Lee, Won Seok Yang

**Affiliations:** Division of Nephrology, Department of Internal Medicine, Asan Medical Center, University of Ulsan College of Medicine, Seoul, Korea

**Keywords:** Acute tubulointerstitial nephritis, anti-neutrophil cytoplasmic antibody, glycosuria, hypokalemia, hypophosphatemia, hypouricemia

## Abstract

**Background:**

Glycosuria is one of the manifestations of acute tubulointerstitial nephritis (ATIN), but may also be observed in other renal diseases. In this study, we investigated the value of non-diabetic glycosuria as a diagnostic clue for ATIN.

**Methods:**

We retrospectively reviewed the medical records of adult patients who underwent a kidney biopsy as an evaluation for serum creatinine > 1.4 mg/dL. Patients with proteinuria in the nephrotic range, diabetes mellitus, or transplanted kidney were excluded. The laboratory abnormalities suggestive of tubular injury were compared between 28 patients (14 men and 14 women, mean age 48.5 ± 14.1 years) with ATIN and 116 patients (76 men and 40 women, mean age 53.1 ± 15.0 years) with other diagnoses.

**Results:**

In ATIN, glycosuria (≥ 1+ on dipstick; 68%) was more frequent than hypophosphatemia (18%), hypouricemia (18%), hypokalemia (18%), and tubular proteinuria (40%). In other diagnoses, glycosuria (≥ 1+) was detected in 7 (6%) patients; 6 of them had the histological diagnosis of antineutrophil cytoplasmic antibody-associated glomerulonephritis. The presence of glycosuria (≥ 1+) had 68% sensitivity and 94% specificity for ATIN, with the positive likelihood ratio of 11.24 and the negative likelihood ratio of 0.34. Pyuria and low total CO_2_ were equally and more sensitive (68% and 71%, respectively) than glycosuria (≥ 1+), but had no diagnostic value due to low specificities (58% and 60%, respectively).

**Conclusion:**

In non-diabetic, non-nephrotic patients undergoing a kidney biopsy for azotemia, 1+ or higher glycosuria, if present, was a good predictor of the diagnosis of ATIN.

## Introduction

Acute tubulointerstitial nephritis (ATIN) is characterized by infiltration of inflammatory cells into the interstitium of the kidneys, which results in injury of tubular epithelial cells. Clinically, it presents with acute kidney injury and can be accompanied by fever, rash, eosinophilia, hematuria, sterile pyuria and/or signs of tubular damage [1,2].

Proximal and distal tubules both can be affected by ATIN. Proximal tubules normally reabsorb small molecular weight proteins, phosphates, uric acids, glucose, and bicarbonate that are filtered through glomerular capillary wall. Injury of proximal tubules, therefore, causes urinary loss of these substances and results in low molecular weight proteinuria, phosphaturia, uricosuria, glycosuria, and normal anion gap metabolic acidosis with hypokalemia [3]. Distal tubules are the sites concentrating urine and excreting acid that is produced though the metabolic process of amino acids, and injury of distal tubules results in nephrogenic diabetes insipidus and distal tubular acidosis [4].

Acute kidney injury caused by ATIN can be reversed by early recognition, elimination of the offending drugs and administration of corticosteroid [1,5]. In several studies, a delay in the initiation of corticosteroid resulted in worse recovery of kidney function [6,7]. Thus, prompt diagnosis is essential, but the specific marker to suspect ATIN is absent and remains to be discovered. In the past, urine eosinophil was considered to be a specific indicator of ATIN, but was found to have no diagnostic value in differentiating ATIN from other renal diseases [8].

In patients with impaired renal function, serum phosphorus, uric acid, potassium and total CO_2_, and urinalysis are usually included in the routine laboratory tests, and hypophosphatemia, hypouricemia, hypokalemia or low total CO_2_ with normal anion gap, if present, may suggest the presence of tubular injury [1].

Non-diabetic glycosuria is a sign of proximal tubular injury [3]. ATIN may cause glycosuria as an isolated tubular defect [9] as well as a global dysfunction of proximal tubules, known as Fanconi syndrome [10]. However, glycosuria may also occur due to the tubular injury associated with other renal diseases including acute tubular necrosis (ATN) [11] and chronic kidney disease [12].

In this study, we investigated the frequency of laboratory abnormalities suggestive of tubular injury in adult ATIN patients, and examined the value of glycosuria as a diagnostic clue for ATIN in the non-diabetic, non-nephrotic patients undergoing a kidney biopsy as an evaluation for azotemia.

## Materials and methods

### Patients

To assess the utility of urine glucose test in identifying ATIN as a cause of elevated creatinine, we performed a retrospective analysis of adult patients who underwent a native kidney biopsy at Asan Medical Center (Seoul, Korea) as a diagnostic evaluation for serum creatinine higher than 1.4 mg/dL. Patients aged <18 years, having diabetes mellitus or transplanted kidney were excluded. The presence of diabetes mellitus was determined by the positive history of diabetes mellitus, a fasting plasma glucose ≥ 126 mg/dL or HbA1c ≥ 6.5% [13]. Glycosuria may also occur in a person who doesn't have diabetes if blood glucose level rises higher than 170–200 mg/dL and the filtered glucose load exceeds the capacity for tubular glucose reabsorption [14]. So, the patients with blood glucose higher than 170 mg/dL on the day when urine glucose was positive were also excluded. Patients with proteinuria in the nephrotic range (24-h urine protein > 3.5 g, urine protein/creatinine >3.5 g/g or urine albumin/creatinine >2.5 g/g [15]) were excluded because ATIN-induced proteinuria is mild and only rarely is in the nephrotic range [1,2]. In some patients with nephrotic syndrome, ATIN was combined to minimal change or focal segmental glomerulosclerosis, and those patients were also excluded because nephrotic syndrome itself was an indication of a kidney biopsy to determine the pathologic type, regardless of the presence of ATIN.

Diagnosis of ATIN was made by the characteristic findings on a kidney biopsy, including interstitial mononuclear cell infiltration, interstitial edema, and tubulitis [16]. On November 2016, our hospital changed the instruments for urinalysis, using the grading scale of the glucose dipstick test different from the previous one, and thus we recruited patients diagnosed before this change. In the period between January 2001 and October 2016, 28 ATIN patients were identified to meet the inclusion criteria. We reviewed the medical records with regard to their demographic data and the laboratory tests performed before and after the kidney biopsy.

To compare the clinical and laboratory features of ATIN with those of other causes of azotemia, we also reviewed the medical records of 116 patients who were not nephrotic but underwent a native kidney biopsy between January 2010 and October 2016 as an evaluation of serum creatinine higher than 1.4 mg/dL and had a histologic diagnosis other than ATIN.

Diabetes was ruled out by past medical history, fasting plasma glucose and HbA1c, but HbA1c was not done in 8 patients of ATIN group and 65 patients of control group, maybe due to the lack of reason to suspect diabetes.

This study was approved by the institutional review board of Asan Medical Center.

### Laboratory parameters

We collected the laboratory data including complete blood count (CBC), serum creatinine, phosphorus, uric acid, potassium, total CO_2_, glucose, HbA1c, urinalysis with microscopy, 24-h urine protein, urine protein/creatinine ratio, and urine albumin/creatinine ratio.

The urine dipstick for glucose has a pad impregnated with glucose oxidase which reacts with glucose in urine to form hydrogen peroxide, causing color change of a chromogen. Urine glucose was graded as follows: negative (not detected), trace (100 mg/dL), 1+ (250 mg/dL), 2+ (500 mg/dL), 3+ (1000 mg/dL), and 4+ (≥ 2000 mg/dL).

Microscopic hematuria and pyuria were defined as red blood cells ≥ 3/high-power field and white blood cells ≥ 3/high-power field, respectively, on microscopic evaluation of urinary sediment [17, 18].

In case of multiple measurements, the highest levels of serum creatinine and urine glucose, the lowest levels of phosphorus, uric acid, potassium and total CO_2_ among the results obtained within 1 month before the kidney biopsy were taken as the representative values. Hypophosphatemia, hypouricemia, hypokalemia and low total CO_2_ were defined as <2.5 mg/dL, <3.0 mg/dL, <3.5 mEq/L and <20 mEq/L, respectively.

Estimated glomerular filtration rate (eGFR) was calculated by Chronic Kidney Disease Epidemiology Collaboration (CKD-EPI) equation [19].

### Statistical analyses

Data were expressed as mean ± standard deviation (SD) for those with normal distribution or median (range) for those with non-normal distribution. Statistical differences between 2 groups were determined by unpaired t-test or the Mann*-*Whitney U test, as appropriate. The categorical variables including fever, rash and eosinophilia in ATIN and control groups were compared by Chi-square test or Fisher’s exact test. The receiver operating characteristic (ROC) curve analysis was used to assess the diagnostic value of glycosuria for the detection of ATIN. Pre-kidney biopsy eGFR was compared with the follow-up eGFR by Wilcoxon Signed Rank test. The statistical analyses were performed using SPSS version 21 (IBM Co., Armonk, NY, USA). *p* values less than .05 were considered statistically significant.

## Results

### Patient characteristics

There were 28 patients (14 men and 14 women) who were diagnosed with ATIN. Their mean age was 48.5 ± 14.1 years (range 20–70 years). ATIN was possibly associated with nonsteroidal anti-inflammatory drugs (*n* = 4), herbal medicine (*n* = 3), antibiotics (*n* = 2), rifampin (*n* = 1), uveitis (*n* = 1) or sarcoidosis (*n* = 1), but the etiology was not clear in the remaining 16 patients.

The control group consisted of 116 patients (76 men and 40 women) who were diagnosed with a disease other than ATIN. Their mean age was 53.1 ± 15.0 years (range, 20–85 years). The pathologic diagnoses of control groups are shown in Table 1.

**Table 1.** Clinical features and laboratory data of the study population.

	ATIN (*n* = 28)	Other Diagnoses (*n* = 116)	*p*-value
Age (years)	48.5 ± 14.1	53.1 ± 15.0	.144
Male/female	14/14	76/40	.128
Fever	8 (29%)	25 (22%)	.428
Rash	5 (18%)	5 (4%)	.024
White blood cells (×10^3^/μL)	8.40 ± 3.17	8.28 ± 3.25	.859
Eosinophilia (>500/μL)	4 (14%)	18 (16%)	1.000
Hemoglobin (g/dL)	9.39 ± 1.85	10.91 ± 2.41	.001
Platelets (×10^3^/μL)	263.7 ± 79.5	253.7 ± 92.9	.601
Creatinine (mg/dL)	3.71 (1.60–16.50)	2.15 (1.42–18.60)	<.001
eGFR (mL/min/1.73m^2^)	17 (4–49)	31 (2–63)	.002
Urine microscopy			
RBC (/HPF)			.072
0–2	14 (50%)	37 (32%)	
≥ 3	14 (50%)	79 (68%)	
WBC (/HPF)			.012
0–2	9 (32%)	68 (59%)	
≥ 3	19 (68%)	48 (41%)	
Pathologic diagnoses			
ATIN	28	–	
IgA nephropathy	–	46	
ANCA–associated GN	–	34	
Focal segmental glomerulosclerosis	–	7	
Hypertensive nephrosclerosis	–	5	
Malignant hypertension	–	3	
Acute tubular necrosis	–	3	
Advanced chronic GN	–	2	
Thrombotic microangiopathy	–	2	
Amyloidosis	–	2	
Others*	–	12	

Data are mean ± standard deviation, median (range) or *n* (%). ANCA: antineutrophil cytoplasmic antibody; ATIN: acute tubulointerstitial nephritis; eGFR: estimated glomerular filtration rate; GN: glomerulonephritis. *Others; Henoch-Schönlein purpura (1), chronic interstitial nephritis (1), lupus nephritis (1), ANCA-negative pauci-immune crescentic GN (1), polyarteritis nodosa (1), warfarin-related nephropathy (1), acute phosphate nephropathy (1), non-diagnostic (5).

### Clinical manifestations and laboratory parameters

Clinical manifestations of ATIN and other diagnoses are also shown in Table 1. There was no significant difference in the frequency of fever, but skin rash was more common in ATIN (18%) than in other diagnoses (4%; *p* = .024).

In CBC, hemoglobin was lower in ATIN (9.39 ± 1.85 g/dL) than in other diagnoses (10.91 ± 2.41 g/dL; *p* = .001), while leukocyte and platelet counts and peripheral eosinophilia were not different between the two groups.

Prebiopsy serum creatinine of ATIN (median 3.71 mg/dL, range 1.60–16.50 mg/dL) was higher than that of other diagnoses (median 2.15 mg/dL, range 1.42–18.60 mg/dL; *p* < .001).

In the urinalysis, pyuria was more common in ATIN (68%) than in other diagnoses (41%; *p* = .012). In ATIN, urine culture was done in 17 of the 19 patients with pyuria and 4 of the 9 patients without pyuria, and all had no significant growth.

### Frequency of glycosuria: ATIN vs other diagnoses

Urinalysis (median) was obtained 2 (range, 1–8) times in the group of ATIN, and 2 (range, 1–7) times in the group of other diagnoses in a period of 1 month prior to the kidney biopsy.

ATIN had a higher frequency of glycosuria as compared with other diagnoses. Urinary glucose was negative in 5 patients, trace in 4 patients, 1+ in 9 patients, 2+ in 6 patients, 3+ in 3 patients and 4+ in 1 patient with ATIN, while it was negative in 99 patients, trace in 10 patients, and 1+ in 7 patients with other diagnoses (*p* < .001) (Table 2).

**Table 2.** Distribution of glycosuria levels in ATIN and other diagnoses.

Urine Glucose on dipstick	ATIN (*n* = 28)	Other Diagnoses (*n* = 116)
Negative	5	99
Trace	4	10
1+	9	7
2+	6	0
3+	3	0
4+	1	0

In ATIN patients with glycosuria ≥ 1+, urine glucose (1+ or higher) was detected 2.11 ± 1.33 times by 2.53 ± 1.78 urinalyses per person. Fourteen of the 19 ATIN patients with glycosuria (≥ 1+) had serum glucose level measured on the day when urine glucose was 1+ or higher, and 8 of the 9 ATIN patients with negative or trace urine glucose had serum glucose level measured on the day when the urinalysis was done. Serum glucose was 107 ± 20 mg/dL in patients with glycosuria (≥ 1+), while it was 99 ± 14 mg/dL in patients with negative or trace urine glucose (*p* = .257). There was no difference in eGFR between those with (*n* = 19, median 13, range 4–46 mL/min/1.73m^2^) and without (*n* = 9, eGFR, median 17, range 7–49 mL/min/1.73m^2^) 1+ or higher glycosuria.

Of the 7 patients with 1+ glycosuria in the group of other diagnoses, 6 had the histological diagnosis of antineutrophil cytoplasmic antibody (ANCA)-associated glomerulonephritis, and the other one had IgA nephropathy. Urine glucose 1+ was detected 1.17 ± 0.41 times by 2.67 ± 1.21 urinalyses per person in the 6 patients with ANCA-associated glomerulonephritis and once by 3 urinalyses in the patient with IgA nephropathy. Serum glucose level measured on the day when urine glucose was detected was 128 ± 17 mg/dL.

### Sensitivity, specificity, positive and negative likelihood ratio of glycosuria for ATIN

**Figure 1.**
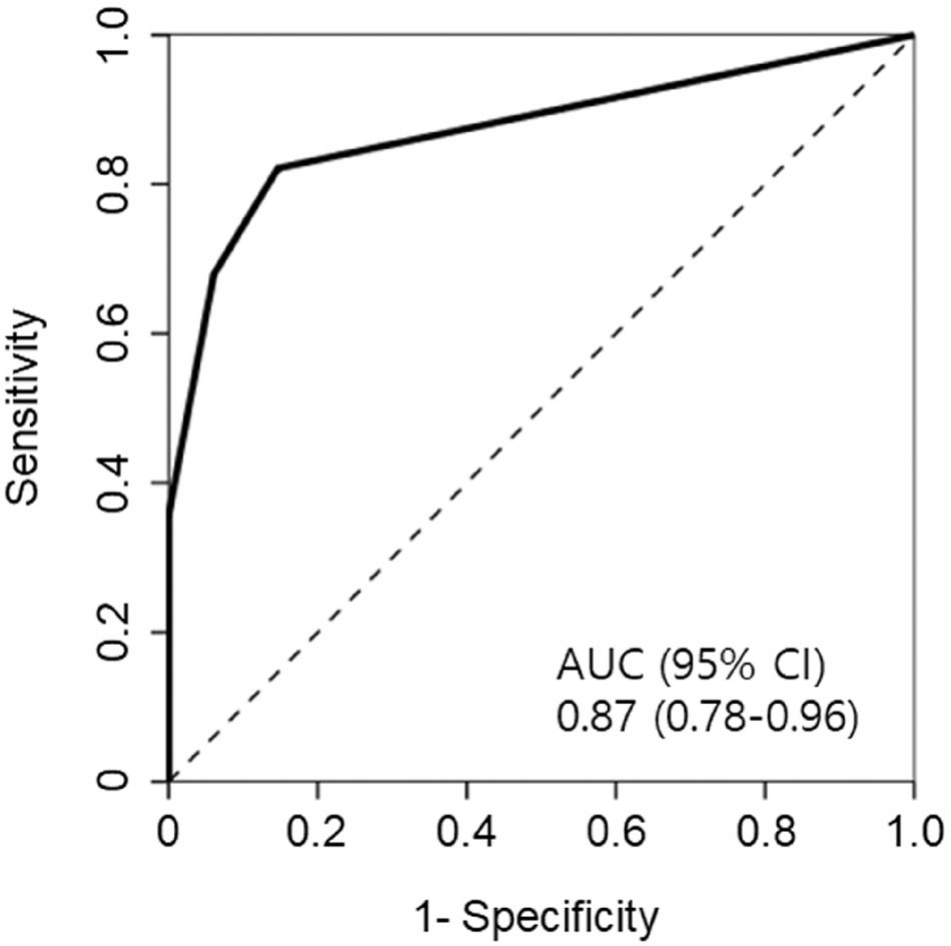
Receiver operating characteristic (ROC) curve for predicting acute tubulointerstitial nephritis from urine dipstick tests for glucose. AUC: the area under the ROC curve; CI: confidence interval.

The area under the ROC curve (AUC) of urine dipstick test for glucose in the prediction of ATIN was 0.87 (95% confidence interval: 0.78–0.96) (Figure 1). Performance of glycosuria to predict ATIN at the trace and 1+ cutoff is shown in Table 3. At the cutoff of trace glycosuria, the presence of glycosuria had 82% sensitivity and 85% specificity for ATIN, with the positive likelihood ratio of 5.61 and the negative likelihood ratio of 0.21. When the cutoff was increased to 1+ for a positive test, the sensitivity declined to 68%, but the specificity increased to 94%, with the positive likelihood ratio of 11.24 and the negative likelihood ratio of 0.34.

**Table 3.** Performance of the different cutoffs for glycosuria to predict ATIN.

Cutoff (Glycosuria)	Sensitivity (95% CI)	Specificity (95% CI)	Positive Likelihood Ratio (95% CI)	Negative Likelihood Ratio (95% CI)
Trace	82 (63–94)	85 (78–91)	5.61 (3.50–8.99)	0.21 (0.09–0.46)
1+	68 (48–84)	94 (88–98)	11.24 (5.25–24.09)	0.34 (0.20–0.59)

### Other laboratory abnormalities suggestive of tubular injury: ATIN vs other diagnoses

Hypophosphatemia, hypouricemia and hypokalemia, the blood chemistries suggestive of tubular injury, were detected in 5 (18%), 5 (18%), 5 (18%) patients, respectively, with ATIN, and in 3 (3%), 1 (1%), 11 (9%) patients, respectively, with the other diagnoses. The difference between the two groups was statistically significant for hypophosphatemia (*p* = .007) and hypouricemia (*p* = .001), but not for hypokalemia (Table 4).

**Table 4.** Blood chemistries suggestive of tubular injury.

	ATIN (*n* = 28)	Other Diagnoses (*n* = 116)	*p*-value
Phosphorus (mg/dL)	3.34 ± 0.82	3.86 ± 1.08	.019
Phosphoru*s* < 2.5 mg/dL	5 (18%)	3 (3%)	.007
Uric acid (mg/dL)	4.53 ± 2.16	7.45 ± 2.15	<.001
Uric acid < 3.0 mg/dL	5 (18%)	1 (1%)	.001
Potassium (mEq/L)	3.91 ± 0.47	4.32 ± 0.67	.003
Potassium< 3.5 mEq/L	5 (18%)	11 (9%)	.311
Total CO_2_ (mEq/L)	18.4 ± 4.2	21.5 ± 5.1	.003
Total CO_2_ < 20 mEq/L	20 (71%)	46 (40%)	.002

Data are mean ± standard deviation or *n* (%).

Low total CO_2_, the parameter suggestive of metabolic acidosis, was also more common in ATIN (71%) than in the other diagnoses (40%; *p* = .002).

Urine samples were submitted to the laboratory for protein electrophoresis in 20 patients with ATIN. In 4 of them, urine protein concentration was too low to perform protein electrophoresis. Accordingly, urine protein electrophoresis was performed in 16 patients, and tubular proteinuria was identified in 8 patients (40% of the patients who submitted urine for protein electrophoresis), in whom glycosuria was negative in 1 patient, 1+ in 2 patients, 2+ in 3 patients, 3+ in 1 patient and 4+ in 1 patient.

### Recovery of renal function in ATIN

After the diagnosis of ATIN was made, twenty-seven patients received steroid in intravenous methylprednisolone pulse therapy (*n* = 2, 500 mg for 3 days followed by oral prednisolone) or oral prednisolone (*n* = 25, 0.76 ± 0.24 mg/kg) with tapering the doses afterwards. In some patients, oral cyclophosphamide was added to the steroid therapy. The remaining 1 patient improved without steroid treatment. Pre-kidney biopsy eGFR was compared with eGFR derived from the last serum creatinine measured within 6 months after the kidney biopsy. The eGFR (median) increased from 17.0 mL/min/1.73m^2^ (range, 4–49) to 51.5 mL/min/1.73m^2^ (range, 11–107) (*p* < .001). Two patients had no improvement of eGFR, but the other 26 patients had an increment of eGFR at least 12 mL/min/1.73m^2^.

## Discussion

In the present study, we evaluated whether the laboratory abnormalities suggestive of tubular injury may serve as a marker for ATIN in patients with elevated serum creatinine, and our data demonstrate that glycosuria in the absence of diabetes mellitus is a valuable clue in suspecting ATIN.

Glycosuria is one of the manifestations of ATIN. In children, there have been several studies that reported the frequency of glycosuria in ATIN, and 5 of 13 (38%) patients [20], 9 of 10 (90%) patients [21], 17 of 25 (68%) patients [22], and 8 of 10 (80%) patients [23], respectively, had glycosuria. In a recent larger study of adult ATIN [24], glycosuria was found in 68% (107/157) of the patients. In these studies, however, it was not stated how glycosuria was defined, and the frequency of glycosuria may vary depending on the definition of glycosuria.

In normal individuals, nearly all glucose filtered through the glomerulus is reabsorbed in the tubules, mainly in the proximal tubules, and glucose appears in the urine at the concentration less than 25 mg/dL [25]. Because glucose concentration measured as ‘trace’ in the dipstick test represents 100 mg/dL, even ‘trace’ glycosuria may indicate abnormal presence of glucose in the urine. In the present study, glycosuria ≥ trace was present in 82% of ATIN patients. In the other renal diseases including glomerular diseases, however, glycosuria may also occur due to the concurrent tubular injury [11, 26], and glycosuria ≥ trace was present in 15% of patients with other diagnoses. Thus, glycosuria ≥ trace was not very specific to ATIN.

So far, the amount of non-diabetic glycosuria that indicates tubular dysfunction has not been well defined, but urine glucose 3+ or higher was suggested as one of the criteria indicating drug-induced tubular dysfunction [27]. In the present study, urine glucose 3+ or higher was observed in only 4 (14%) of 28 patients with ATIN, and was not sensitive in detecting ATIN. At the cutoff of 1+, glycosuria had 68% sensitivity and 94% specificity for ATIN, with the positive likelihood ratio of 11.24. The latter indicates about 11-fold increase in the odds of having ATIN if a patient has a positive test result [28]. Thus, even 1+ glycosuria detected on urine dipstick significantly increases the probability of ATIN. Of the other diagnoses, glycosuria was detected mainly in ANCA-associated glomerulonephritis in which interstitial nephritis is often found in addition to crescentic glomerulonephritis [29]. The frequency of 1+ glycosuria was 18% (6/34) in ANCA-associated glomerulonephritis. Our data suggest that if those patients are excluded by ANCA testing, non-diabetic glycosuria can be further specific for ATIN.

Non-diabetic glycosuria can also be observed in ATN. The proximal tubules are subdivided into S1, S2 and S3 segments. Normally, 90% of filtered glucose is reabsorbed in the S1 and S2 segments and the remaining 10% is reabsorbed in the S3 segment [30]. In ischemic ATN, the most severe tubular injury takes place in the S3 segment [31]. Of the nephrotoxins, aminoglycosides affect the S1 and S2 segments, whereas cisplatin affects the S3 segment [32]. The resulting tubular injuries may cause glycosuria as well as azotemia [11]. In patients with suspected ischemic or nephrotoxic ATN, however, the diagnosis is made clinically and kidney biopsy is usually not performed. Thus, ATN was not a significant cause of glycosuria in the present study where the patients underwent a kidney biopsy for azotemia of uncertain etiology.

A low level of serum phosphorus, uric acid or potassium in patients with azotemia also suggests the possibility of ATIN [1]. In the present study, the frequency of hypophosphatemia, hypouricemia or hypokalemia in ATIN was much lower than that of glycosuria. It may be because decreased glomerular filtrations of phosphorus, uric acid and potassium due to the accompanying acute kidney injury in ATIN may mask urinary losses of phosphorus, uric acid and potassium. ATIN may also cause an isolated tubular defect with benign reversible glycosuria [9] rather than global dysfunction of proximal tubules known as Fanconi syndrome.

In proximal tubular injury, low molecular weight proteins filtered through glomeruli are not reabsorbed and appear in the urine. The tubular proteinuria can be identified in protein electrophoresis by its characteristic pattern [33], and can be a clue for ATIN. In this study, some of the samples had too low protein concentration for electrophoresis, and tubular proteinuria was detected in 8 (40%) of 20 patients who submitted urine for the test. Thus, the sensitivity of urine protein electrophoresis in the detection of ATIN was lower than that of urine glucose test. However, there was a patient who had no glycosuria, but had a positive result of tubular proteinuria. So, urine protein electrophoresis may be used in conjunction with urine glucose to improve the sensitivity in the diagnosis of ATIN.

Pyuria and low total CO_2_ were equally and more sensitive (68% and 71%, respectively) than glycosuria (≥ 1+), but had no diagnostic value due to low specificities (58% and 60%, respectively).

Clinical suspicion of ATIN is important for early diagnosis, but it is not easy due to lack of a sensitive and specific marker for ATIN. Recently, patients with ATIN were shown to have higher levels of urine TNF-α and IL-9 than those with other diagnoses, and inclusion of urinary TNF-α and IL-9 was suggested to improve pre-biopsy diagnosis of ATIN [34]. AUC is a measure of the overall performance of a test in the diagnosis of a disease [35]. Measurements of urinary TNF-α and IL-9 improved AUC over clinicians’ prebiopsy diagnosis (0.84 vs. 0.62). As compared with the urinary cytokine measurements, the dipstick test for urine glucose has several advantages. It is simple and much cheaper, and routinely performed in patients with azotemia. In addition, AUC of renal glycosuria to predict ATIN was 0.87 which is not inferior to that of combined tests of urine TNF-α and IL-9.

Our study has potential limitations. First, the sample size is small, and so the present study did not cover ATINs of diverse etiologies. Second, the urinary dipstick measurement of glucose is semi-quantitative, and the results can be affected by the level of urine concentration. Quantitative measurements such as urine glucose/creatinine ratio or fractional excretion of glucose could be more appropriate to define glycosuria. Third, the glucose dipstick test may have false positives and false negatives. For example, it can be false negative in patients taking ascorbic acid which interferes with the reaction for glucose detection in the dipstick test. Commercial soft drinks or green tea may contain a large amount of ascorbic acid as an antioxidant [36]. So, ascorbic acid or ascorbic acid-containing beverage should be avoided before obtaining samples for urinalysis, but it was not checked in our study population. Fourth, there may be a selection bias as it is a retrospective study. If renal glycosuria was present, it is more likely to make a decision to do a kidney biopsy, which may result in the inclusion of ATIN patients with glycosuria more than those without it.

In conclusion, glycosuria was more common in ATIN than in other renal pathologies. In non-diabetic, non-nephrotic patients undergoing a kidney biopsy for azotemia, 1+ or higher glycosuria, if present, was a good predictor of the diagnosis of ATIN. Due to the retrospective design of this study, our findings need further validation in future prospective studies.

## References

[1] Raghavan R, Eknoyan G. Acute interstitial nephritis – a reappraisal and update. Clin Nephrol. 2014;82(3):149–162. 25079860 10.5414/CN108386 PMC4928030

[2] Praga M, González E. Acute interstitial nephritis. Kidney Int. 2010;77(11):956–961. 20336051 10.1038/ki.2010.89

[3] Foreman JW. Fanconi Syndrome. Pediatr Clin North Am. 2019;66(1):159–167. 30454741 10.1016/j.pcl.2018.09.002

[4] Vigeral P, Kanfer A, Kenouch S, et al. Interstitial nephropathies induced by meticillin: demonstration of distal tubule functional anomalies. Ann Med Interne. 1987;138(8):631–634. 3450212

[5] Prendecki M, Tanna A, Salama AD, et al. Long-term outcome in biopsy-proven acute interstitial nephritis treated with steroids. Clin Kidney J. 2017;10(2):233–239. 28396740 10.1093/ckj/sfw116 PMC5381232

[6] Fernandez-Juarez G, Perez JV, Caravaca-Fontán F, et al. Duration of treatment with corticosteroids and recovery of kidney function in acute interstitial nephritis. Clin J Am Soc Nephrol. 2018;13(12):1851–1858. 30397027 10.2215/CJN.01390118 PMC6302327

[7] Muriithi AK, Leung N, Valeri AM, et al. Biopsy-proven acute interstitial nephritis, 1993–2011: a case series. Am J Kidney Dis. 2014;64(4):558–566. 24927897 10.1053/j.ajkd.2014.04.027

[8] Muriithi AK, Nasr SH, Leung N. Utility of urine eosinophils in the diagnosis of acute interstitial nephritis. Clin J Am Soc Nephrol. 2013;8(11):1857–1862. 24052222 10.2215/CJN.01330213 PMC3817898

[9] Limsuwat C, Prabhakar SS. Reversible renal glycosuria in acute interstitial nephritis. Am J Med Sci. 2012;344(3):245–247. 22929613 10.1097/MAJ.0b013e318254bd71

[10] Neelakantappa K, Gallo GR, Lowenstein J. Ranitidine-associated interstitial nephritis and Fanconi syndrome. Am J Kidney Dis. 1993;22(2):333–336. 8352262 10.1016/s0272-6386(12)70327-x

[11] Hall AM, Bass P, Unwin RJ. Drug-induced renal Fanconi syndrome. QJM. 2014;107(4):261–269. 24368854 10.1093/qjmed/hct258

[12] Hung CC, Lin HY, Lee JJ, et al. Glycosuria and renal outcomes in patients with nondiabetic advanced chronic kidney disease. Sci Rep. 2016;6:39372. 28008953 10.1038/srep39372 PMC5180243

[13] American Diabetes Association. Classification and diagnosis of diabetes: standards of medical care in diabetes-2018. Diabetes Care. 2018;41(Suppl 1):S13–S27. 29222373 10.2337/dc18-S002

[14] Osaki A, Okada S, Saito T, et al. Renal threshold for glucose reabsorption predicts diabetes improvement by sodium-glucose cotransporter 2 inhibitor therapy. J Diabetes Investig. 2016;7(5):751–754. 10.1111/jdi.12473 PMC5009138 27181936

[15] Hovind P, Rossing P, Tarnow L, et al. Remission of nephrotic-range albuminuria in type 1 diabetic patients. Diabetes Care. 2001;24(11):1972–1977. 11679467 10.2337/diacare.24.11.1972

[16] Fogo AB, Lusco MA, Najafian B, et al. AJKD atlas of renal pathology: acute interstitial nephritis. Am J Kidney Dis. 2016;67(6):e35–e36. 27211376 10.1053/j.ajkd.2016.04.002

[17] Davis R, Jones JS, Barocas DA, et al. American urological association. diagnosis, evaluation and follow-up of asymptomatic microhematuria (AMH) in adults: AUA guideline. J Urol. 2012;188(6 Suppl):2473–2481. 23098784 10.1016/j.juro.2012.09.078

[18] Wise GJ, Schlegel PN. Sterile pyuria. N Engl J Med. 2015;372(11):1048–1054. 25760357 10.1056/NEJMra1410052

[19] Levey AS, Stevens LA, Schmid CH, et al. A new equation to estimate glomerular filtration rate. Ann Intern Med. 2009;150(9):604–612. 19414839 10.7326/0003-4819-150-9-200905050-00006 PMC2763564

[20] Ellis D, Fried WA, Yunis EJ, et al. Acute interstitial nephritis in children: a report of 13 cases and review of the literature. Pediatrics. 1981;67(6):862–870. 7015263

[21] Koskimies O, Holmberg C. Interstitial nephritis of acute onset. Arch Dis Child. 1985;60(8):752–755. 4037860 10.1136/adc.60.8.752 PMC1777412

[22] Clavé S, Rousset-Rouvière C, Daniel L, et al. Acute tubulointerstitial nephritis in children and chronic kidney disease. Arch Pediatr. 2019;26(5):290–294. 31281039 10.1016/j.arcped.2019.05.002

[23] Roy S, Awogbemi T, Holt RCL. Acute tubulointerstitial nephritis in children- a retrospective case series in a UK tertiary paediatric centre. BMC Nephrol. 2020;21(1):17. 31937254 10.1186/s12882-020-1681-7 PMC6961306

[24] Su T, Gu Y, Sun P, et al. Etiology and renal outcomes of acute tubulointerstitial nephritis: a single-center prospective cohort study in China. Nephrol Dial Transplant. 2018;33(7):1180–1188. 28992223 10.1093/ndt/gfx247

[25] Cowart SL, Stachura ME. Chapter 139. Glucosuria. In: Walker HK, Hall WD, Hurst JW, editors. Clinical methods: the history, physical, and laboratory examinations. 3rd ed. Boston: Butterworths; 1990. 21250045

[26] Woronik V, Freitas IF, Saldanha LB, et al. Glycosuria in glomerular diseases: histopathology and clinical correlations. Braz J Med Biol Res. 1998;31(5):633–637. 9698767 10.1590/s0100-879x1998000500005

[27] Mehta RL, Awdishu L, Davenport A, et al. Phenotype standardization for drug-induced kidney disease. Kidney Int. 2015;88(2):226–234. 25853333 10.1038/ki.2015.115 PMC4758130

[28] Akobeng AK. Understanding diagnostic tests 2: likelihood ratios, pre- and post-test probabilities and their use in clinical practice. Acta Paediatr. 2007;96(4):487–491. 17306009 10.1111/j.1651-2227.2006.00179.x

[29] Zonozi R, Niles JL, Cortazar FB. Renal involvement in antineutrophil cytoplasmic antibody-associated vasculitis. Rheum Dis Clin North Am. 2018;44(4):525–543. 30274621 10.1016/j.rdc.2018.06.001

[30] Faillie JL. Pharmacological aspects of the safety of gliflozins. Pharmacol Res. 2017;118:71–81. 27389050 10.1016/j.phrs.2016.07.001

[31] Bonventre JV, Yang L. Cellular pathophysiology of ischemic acute kidney injury. J Clin Invest. 2011;121(11):4210–4221. 22045571 10.1172/JCI45161 PMC3204829

[32] Cristofori P, Zanetti E, Fregona D, et al. Renal proximal tubule segment-specific nephrotoxicity: an overview on biomarkers and histopathology. Toxicol Pathol. 2007;35(2):270–275. 17366321 10.1080/01926230601187430

[33] Smith ER, Cai MM, McMahon LP, et al. The value of simultaneous measurements of urinary albumin and total protein in proteinuric patients. Nephrol Dial Transplant. 2012;27(4):1534–1541. 22193048 10.1093/ndt/gfr708

[34] Moledina DG, Wilson FP, Pober JS, et al. Urine TNF-α and IL-9 for clinical diagnosis of acute interstitial nephritis. JCI Insight. 2019;4(10):pii:127456. 10.1172/jci.insight.127456 PMC6542610 31092735

[35] Park SH, Goo JM, Jo CH. Receiver operating characteristic (ROC) curve: practical review for radiologists. Korean J Radiol. 2004;5(1):11–18. 15064554 10.3348/kjr.2004.5.1.11 PMC2698108

[36] Matsuo C, Harashima N, Sekine K, et al. [Influence of commercial soft drinks or green tea intake to occult blood and sugar tests with urinalysis reagent strips]. Rinsho Byori. 2009;57(9):834–841. 19860208

